# Clinical significance and immune infiltration analyses of a novel coagulation-related signature in ovarian cancer

**DOI:** 10.1186/s12935-023-03040-3

**Published:** 2023-10-06

**Authors:** Jiani Yang, Chao Wang, Yue Zhang, Shanshan Cheng, Meixuan Wu, Sijia Gu, Shilin Xu, Yongsong Wu, Jindan Sheng, Dominic Chih-Cheng Voon, Yu Wang

**Affiliations:** 1 Department of Gynecology, Shanghai First Maternity and Infant Hospital, School of Medicine, Tongji University, Shanghai, China; 2 Shanghai Key Laboratory of Maternal Fetal Medicine, Shanghai Institute of Maternal-Fetal Medicine and Gynecologic Oncology, Shanghai First Maternity and Infant Hospital, School of Medicine, Tongji University, Shanghai, 200092 China; 3 Department of Obstetrics and Gynecology, Renji Hospital, School of Medicine, Shanghai Jiaotong University, Shanghai, China; 4 Cancer Research Institute, Kanazawa University, Kanazawa, Ishikawa 9201192 Japan; 5 Institute of Frontier Sciences Initiative, Kanazawa University, Kanazawa, Ishikawa 9201192 Japan

**Keywords:** Coagulation, Immune microenvironment, Ovarian cancer, Prognosis signature

## Abstract

Ovarian cancer (OV) is the most lethal gynecological malignancies worldwide. The coagulation cascade could induce tumor cell infiltration and contribute to OV progression. However, coagulation-related gene (CRG) signature for OV prognosis hasn’t been determined yet. In this study, we evaluated the prognostic value of coagulation scores through receiver operating characteristics (ROC) analysis and K-M curves, among OV patients at our institution. Based on the transcriptome data of TCGA-OV cohort, we stratified two coagulation-related subtypes with distinct differences in prognosis and tumor immune microenvironment (p < 0.05). Moreover, from the 6406 differentially-expressed genes (DEGs) between the GTEx (n = 180) and TCGA-OV cohorts (n = 376), we identified 138 potential CRGs. Through LASSO-Cox algorithm, we finally distinguished a 3-gene signature (SERPINA10, CD38, and ZBTB16), with promising prognostic ability in both TCGA (p < 0.001) and ICGC cohorts (p = 0.040). Stepwise, we constructed a nomogram based on the clinical features and coagulation-related signature for overall survival prediction, with the C-index of 0.6761, which was evaluated by calibration curves. Especially, based on tissue microarrays analysis, Quantitative real-time fluorescence PCR (qRT-PCR), and Western Blot, we found that aberrant upregulation of CRGs was related to poor prognosis in OV at both mRNA and protein level (p < 0.05). Collectively, the coagulation-related signature was a robust prognostic biomarker, which could provide therapeutic benefits for chemotherapy/immunotherapy and assist clinical decision in OV patients.

**Supplementary Information:**

The online version contains supplementary material available at 10.1186/s12935-023-03040-3.

## Introduction

Ovarian cancer (OV) is one of fatal gynecological malignancies worldwide, which threatens women’s safety and health [1]. There were 19,710 new cases and 13,270 deaths related to OV in the United States, which was estimated for 2023 [2]. Due to the lack of specific symptoms and signs, approximately 70% OV patients were diagnosed at advanced stages, which could lead to a poor 5-year overall survival (OS) rate of 30% [3, 4]. After the initial therapy of surgery followed with platinum-based chemotherapy, almost 70% OV patients finally suffer tumor recurrence [5]. Accordingly, there is a pressing urgency to identify appropriate prognostic biomarkers, so as to carry out personalized treatment.

Coagulation, one of the hallmarks of tumor, could be a consequence of increasing plasma extravasation and vascular permeability which leads to extravascular coagulation，or be activated by disruption of vessels which leads to intravascular coagulation [6]. Patients with malignant tumors are prone to develop coagulation disorders, including cancer-associated thrombosis (CAT) [7]. Accumulating evidence shows that tumor cells could release procoagulant factors, such as tissue factors, which might trigger coagulation cascades [8]. On the other hand, tumor coagulum, a cancer-driven network of molecular effectors favoring bleeding or thrombosis, could interact with the tumor microenvironment (TME) to orchestrate cancer inhibition or progression [9]. Accordingly, anticoagulants could be an effective adjuvant treatment to the Immune Checkpoint Blockers (ICB) therapy to boost antitumor immunity, which have been validated in malignant melanomas [10]. As for OV, our recent research indicated that OV patients with the disorder of coagulation system suffered poor prognosis [11], though the role of coagulation in OV was still not clearly understood yet.

Therefore, in this study, we used bioinformatics algorithms to assess the relevance of coagulation with TME in OV. Based on the TCGA cohort, we identified the coagulation-related molecular subtypes through the unsupervised clustering algorithm, and compared the TME and immunotherapy response. We further filtered differential CRGs significantly associated with OV patient prognosis and constructed a 3-gene prognostic model (SERPINA10, CD38, and ZBTB16), which could provide a promising candidate tool to predict OV prognosis and facilitate clinical management.

## Methods

### Patient selection and data collection

**Fig. 1:**
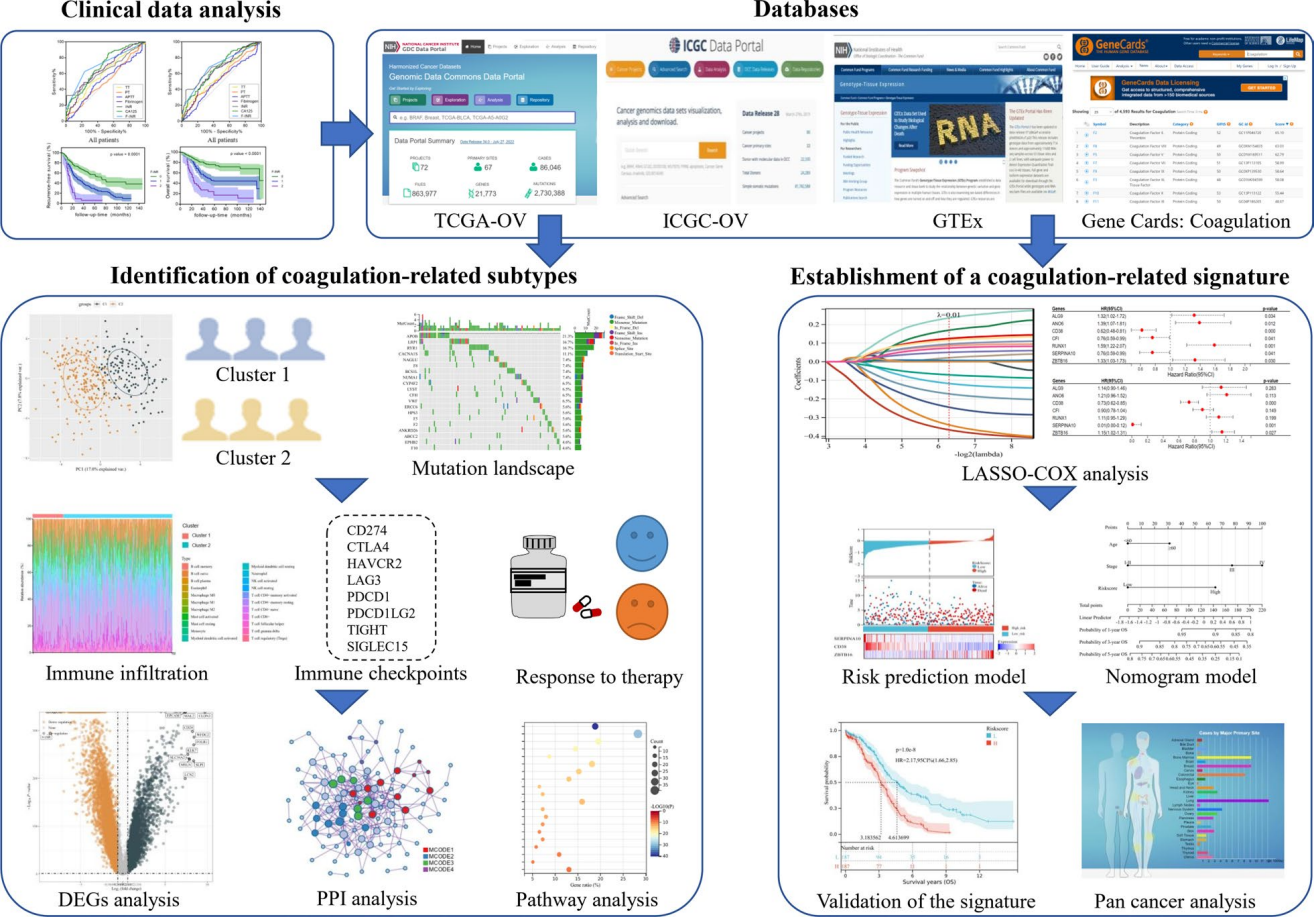
The overall flowchart of the research. DEGs, differentially-expressed genes; PPI, protein–protein interaction

**Fig. 2:**
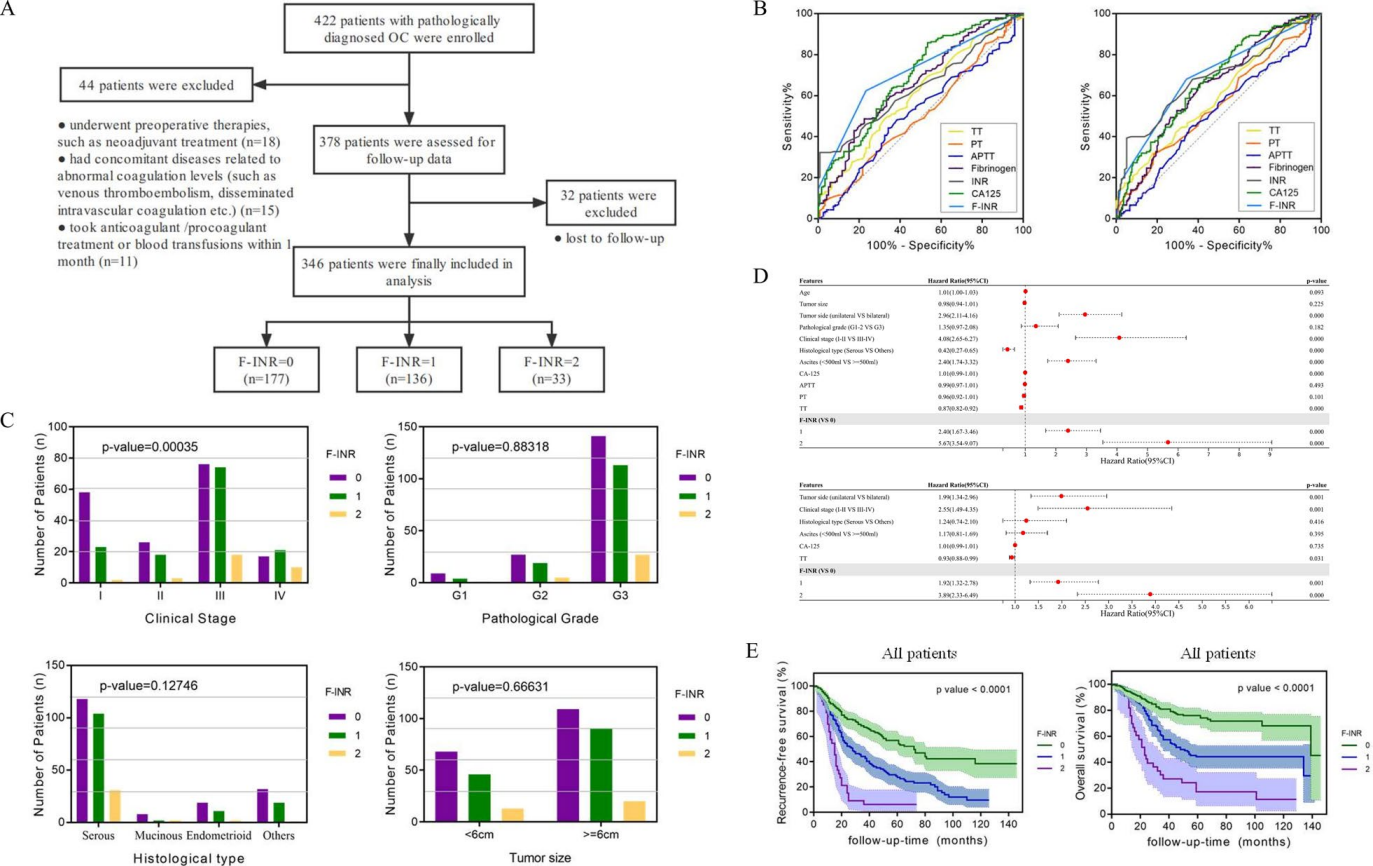
Clinical significance of coagulation indexes in ovarian cancer (OV) prognosis. **A** The flowchart of OV patient enrollment. **B** The Receiver operating characteristic (ROC) curves of coagulation variables including thrombin time (TT), prothrombin time (PT), activated partial thromboplastin time (APTT), fibrinogen, international normalized ratio (INR), and the combination of fibrinogen and international normalized ratio (F-INR) for predictive recurrence-free survival (RFS, left) and overall survival (OS, right) among OV patients. **C** The relationship between F-INR and the International Federation of Gynecology and Obstetrics stage (FIGO stage, left, up), pathological grade (right, up), histological type (left, bottom), and tumor size (right, bottom). **D** The univariate (up) and multivariate (bottom) Cox Hazards Regression analysis for OS in OV patients. **E**. The Kaplan–Meier survival curves classified by the F-INR for RFS (left) and OS (right) among all OV patients. HR, hazard ratio; 95% CI 95% confidence interval

The overall flowchart of the research was shown in Fig. 1. We retrospectively reviewed data from 422 OV patients who underwent surgery at Renji Hospital Affiliated to Shanghai Jiaotong University School of Medicine between June 2008 and January 2018. The criteria for inclusion were: (1) no co-existing or prior cancers within 5 years; (2) histologically confirmed OV; (3) underwent standard operation aimed to achieve optimal tumor debulking followed by platinum-based chemotherapy; and (4) with available clinical data. Patients were excluded from our study if they: (1) underwent preoperative therapies, such as neoadjuvant treatment (n = 18); (2) had concomitant diseases related to abnormal coagulation levels (including venous thromboembolism, disseminated intravascular coagulation etc.) (n = 15); (3) took anticoagulant/ procoagulant treatment (n = 11); (4) were lost to follow-up (n = 32). Finally, 346 patients were involved in our research (Fig. 2A).

Patient demographics, including age, tumor size, histologic grade, and clinical stage were collected from medical records at our institution. The blood tests for coagulation indexes were conducted 1 day before operation. The Ethics Committee of the Renji Hospital Affiliated to Shanghai Jiaotong University School of Medicine approved the research, while all patients could provide informed consents for the usage of their information on research purpose.

### Publicly available datasets and preprocessing

Based on the GeneCards website (https://www.genecards.org/), we retrieved Coagulation-related genes (CRGs, Relevance Score ≥ 3) via searching the term “coagulation”. We have downloaded both RNA-sequencing (RNA-seq) data and corresponding clinical characteristics from the Cancer Genome Atlas website (https://portal.gdc.com; TCGA) as the training cohort, and the International Cancer Genome Consortium website (https://dcc.icgc.org; ICGC) as the validation cohort. Meanwhile, we downloaded transcriptome data of normal tissues from the Genotype-Tissue Expression database (https://gtexportal.org; GTEx) as controls. We converted the probes into gene symbols through the corresponding platform annotation file and normalized the raw count data through the limma package (R software, version 3.36.2). According to the adjusted P < 0.05 and cut-off criteria of | Log2 (Fold Change) |> 1, we filteredthe differentially-expressed genes (DEGs) between OC tissues and controls. Moreover, we evaluated the underlying functions of the potential DEGs through Gene Ontology (GO) and the Kyoto Encyclopedia of Genes and Genomes (KEGG) pathway enrichment analysis.

### Identification of coagulation subtypes and somatic mutation analysis

Based on the Genecards dataset (https://www.genecards.org), we defined 373 CRGs with Relevance Score ≥ 3. We applied the unsupervised clustering Pam method of Euclidean and Ward’s linkage, so as to identify coagulation-related. We defined the coagulation subtypes using the “ConsensusClusterPlus” package in R software and repeated the procedure 100 times to ensure classification stability. Stepwise, we performed the Principal component analysis (PCA) to visualize distribution difference of the coagulation-related subtypes. We also evaluated the relationships between the coagulation subtypes and clinical features such as age, clinical stage, pathological grade, and histologic type, which were visualized the association by the Sankey diagram, using the “ggalluvial” package in R software. We applied the Kaplan–Meier survival curves analysis to compare prognosis of different clusters in the TCGA-OV dataset. The data of somatic mutations were downloaded from Genomic Data Commons and visualized using the “maftools” package in R software. The Oncoplot showed the somatic landscape of OV cohort, stratified by coagulation subtype.

### Analysis of immune landscape and drug sensitivity

In order to provide a brief view of the immune microenvironment, we verified the abundance proportion of 22 typical tumor-infiltrating immune cells through the CIBERSORT algorithm (https://cibersortx.stanford.edu/) [12]. To predict patient response towards immunotherapy, we analyzed the relationship between the coagulation-related signature and immune checkpoint genes expression, including CTLA4, CD274, LAG3, HAVCR2, PDCD1LG2, PDCD1, SIGLEC15, and TIGIT, through the Pearson's test. According to the TIDE datasets (http://tide.dfci.harvard.edu), we evaluated the Potential immune checkpoint blockade (ICB) response of OV patients.

Additionally, in order to evaluate patient response towards chemotherapy, we downloaded the drug response data and genomic markers of drug sensitivity from the Genomics of Drug Sensitivity in Cancer datasets (https://www.cancerrxgene.org, GDSC), one of the largest public pharmacogenomics database worldwide. Next, we conducted the prediction for half-maximal inhibitory concentration values (IC50) through the Ridge Regression, which was performed via the "pRRophetic" package of R software.

### Construction and validation of coagulation-related signature

We filtered the differentially-expressed genes (DEGs) between OV tissues and controls, with | Log2 (Fold Change) |> 1 and the adjusted P < 0.05. Then, we identified differentially expressed coagulation-related genes (DE-CRGs) through Venn diagram. To evaluate prognosis value of the identified DE-CRGs, the Kaplan–Meier (K–M) curves were applied. Stepwise, in order to filter prognostic CRGs for signature construction, we performed the Least Absolute Shrinkage and Selection Operator (LASSO)—COX Regression algorithm with tenfold cross-validation, using the "glmnet" package of R software. Through the "timeROC" package of R software, we also conducted the ROC analysis for 1-year, 3-year, and 5-year survival rate of patients. Furthermore, in order to select independent risk factors of OV prognosis, we performed both uni-variate and multi-variate Cox Regression analyses. Based on the selected variables, we then constructed a nomogram for 1-year, 3-year, and 5-year prognosis through the “rms” package of R software.

### Immunohistochemistry evaluation

For the immunohistochemistry (IHC) analysis, tissue samples were de-waxed, hydration, and wash. After microwave antigen retrieval procedure, the sections were then treated with 3% H_2_O_2_ for blockage of endogenous peroxidase activity. The sections were microwaved for antigen retrieval, and then treated with 3% H2O2 for endogenous peroxidase blockage. Stepwise, the slides were sequentially incubated overnight with Anti- ZBTB16 antibody (ABclonal, A5863, 1:50) and horseradish peroxidase (HRP)—conjugated secondary antibody (Abclonal, AS014). Then, we visualized and counter-stained the signals by diaminobenzidine and hematoxylin. Without information about patients, 2 experienced pathologists scored the signal intensity and percent of IHC slides independently. The staining intensity was graded on a four-tier scale, from 0 to 3 (0 = absent, 1 = weak, 2 = moderate, and 3 = strong). The histochemistry score (H-score) was determined semi-quantitatively based on the staining intensity and proportion of labeled cells: H-Score = 1* percent of weak intensity cells + 2* percent of moderate intensity cells + 3* percent of strong intensity cells. Higher H-score was defined as higher protein expression (maximum score, 300) [13].

### RT-PCR analysis

Following the manufacturer’s instructions, the total RNA from tissues was extracted through Trizol Reagent (Merk, T9424) and then reverse transcribed into cDNA though the RevertAid First Strand cDNA Synthesis Kit (Thermo Fisher Scientific, K1622). Stepwise, we conducted the polymerase chain reaction (PCR) analysis via the SYBR Green Master Mix (Thermo Fisher Scientific, A25742). All the reactions were repeated for at least 3 times. We designed the primer sequences as follows: GAPDH, Forward: 5′-GGCAAATTCCATGGCACCG -3′ and Reverse: 5′- TCGCCCCACTTGATTTTGGA -3′; ZBTB16, Forward: 5′-GAGATCCTCTTCCACCGCAAT -3′ and Reverse: 5′-CCGCATACAGCAGGTCATC -3′; CD38, Forward: 5′-AGACTGCCAAAGTGTATGGGA -3′ and Reverse: 5′ -GCAAGGTACGGTCTGAGTTCC; SERPINA10, Forward: 5′-TCTTTAAGGGACTCAGAGAGACC -3′ and Reverse: 5′-TGTGAGGCATTGCGAAAATTCA. GAPDH was set as an internal control. The comparative expression level was evaluated by 2-ΔΔCt method.

### Western blot analysis

Total protein of tissues was extracted through the ice-cold radioimmunoprecipitation lysis buffer (RIPA, Thermo Fisher Scientific, 89,900), which contained the protease inhibitor cocktail (Merk, P8340). Subsequently, the extracted proteins were quantified through the BCA Assay Kit (Beyotime, P0010) and boiled for degeneration. Then, we separated the proteins in SDS-PAGE (Yeasen, 20315ES05) and transferred them into the PVDF membrane (Merk, 3,010,040,001). After being blocked into 5% Bovine serum albumin (BSA, Yeasen, 36104ES25), the PVDF membrane was then incubated with primary antibodies: Anti-beta-actin (Proteintech, 20,536, 1:1000), Anti- ZBTB16 antibody (ABclonal, A5863, 1:1000), Anti-SERPINA10 antibody (ABclonal, A7106, 1:1000), and Anti-CD38 antibody (ABclonal, A1680, 1:1000). Stepwise, the membranes were incubated in secondary antibodies: Goat Anti-Mouse IgG (Proteintech, SA00001-1, 1:1000) and Goat Anti-Rabbit IgG (ABclonal, AS014, 1:1000), followed by enhanced chemiluminescence to display bands.

### Statistical analysis

We evaluated differences of continuous and categorical variables through T-test and Chi-square test, respectively. We determined prognostic factors using both univariate and multivariate analyses through the Cox's Hazards Regression classifier. Stepwise, survival curves were graphed by Kaplan–Meier methods and compared via the Log-rank test. The ROC curve was applied and the area under the curve (AUC) were evaluated among coagulation indexes. All bioinformatic statistical analyses were conducted by the R software (version 4.0.3). The P < 0.05 was defined as statistically significant for all applied tests.

## Results

### Clinical significance of coagulation indexes in ovarian cancer prognosis

According to the inclusion and exclusion criteria, a total of 346 OV patients were finally involved in the study (Fig. 2A). The clinicopathological features of OV patients were listed in Table 1. The median and mean follow-up time for patients was 50 months (range, 28–73 months) and 54.58 ± 33.17 months. According to the ROC curves of coagulation variables, compared to activated partial thromboplastin time (APTT), prothrombin time (PT), and thrombin time (TT), fibrinogen and International normalized ratio (INR) had superior predictive value, with the area under curve (AUC) of 0.658 (95% CI 0.598–0.718) and 0.643 (95% CI 0.585–0.670) for RFS; 0.640 (95% CI 0.582–0.698) and 0.684 (95% CI 0.626–0.742) for OS ( Fig. 2B). Based on the Youden index, the cut-off values were set at 3.95 g/L for fibrinogen and 0.84 for INR.

**Table 1:** Clinicopathological features of 346 ovarian cancer (OV) patients

Features	Without recurrence (n = 120)	With recurrence (n = 226)	p-value
Age (years)	56.18 ± 9.52	59.19 ± 11.13	0.060
Tumor size (cm)	7.75 ± 4.51	7.65 ± 4.89	0.559
Tumor side, n (%)			0.000
Unilateral	87 (72.5%)	99 (44.0%)	−
Bilateral	33 (27.5%)	127 (56.0%)	−
Pathological grade, n (%)			0.886
G1–2	23 (19.2%)	42 (18.6%)	−
G3	97 (80.8%)	184 (81.4%)	−
Clinical stage, n (%)			0.000
I–II	75 (62.5%)	55 (24.3%)	−
III–IV	45 (37.5%)	171 (75.7%)	−
Histological type, n (%)			0.003
Serous	73 (60.8%)	177 (78.3%)	−
Mucinous	4 (3.3%)	8 (3.5%)	−
Endometrioid	18 (15.0%)	14 (6.2%)	−
Others	25 (20.8%)	27 (11.9%)	−
Ascites, n (%)			0.000
< 500 ml	98 (81.7%)	121 (53.5%)	−
> 500 ml	22 (18.3%)	105 (46.5%)	−
CA-125 (U/mL)	552.17 ± 844.30	1403.96 ± 1784.31	0.000
Fibrinogen (g/L)	3.45 ± 1.43	4.49 ± 2.35	0.002
APTT (s)	27.51 ± 6.43	26.93 ± 7.34	0.148
PT (s)	13.23 ± 10.35	12.23 ± 4.37	0.029
TT (s)	16.40 ± 2.01	15.37 ± 2.79	0.005
INR	1.01 ± 0.13	0.89 ± 0.20	0.000
F-INR, n (%)			0.000
F-INR = 0	92 (76.7%)	85 (37.6%)	−
F-INR = 1	28 (23.3%)	108 (47.8%)	−
F-INR = 2	0 (0.00%)	33 (14.6%)	−

*APTT* Activated partial thromboplastin time, *PT* Prothrombin time, *TT* thrombin time, *INR* International normalized ratio, *F-INR* The combination of fibrinogen and INR

Patients were then classified into three F-INR score groups referring to the cut-off values as following: F-INR score = 2 (fibrinogen ≥ 3.95 g/L and INR < 0.84), F-INR score = 1 (fibrinogen >  = 3.95 g/L or INR < 0.84), and F-INR score = 0 (fibrinogen < 3.95 g/L and INR >  = 0.84). The AUC value for the F-INR scoring was 0.712 (95% CI 0.658– 0.767) and 0.692 (95% CI 0.636–0.749) for RFS and OS, respectively. The correlation between F-INR and other clinicopathological features was presented in Table 1. We found that patients with higher F-INR score had more advanced FIGO stage (p = 0.00035, Fig. 2C). Then, through univariate and multivariate analyses, we determined that besides tumor side and FIGO stage, F-INR (HR 3.89; 95% CI 2.33–6.49; p = 0.000) was also an independent prognostic factor for OV patients ( Fig. 2D). The K–M curves (Fig. 2E) indicated that F-INR score was significantly associated with both RFS (p < 0.0001) and OS (p < 0.0001).

### Identification of coagulation-related subtypes and somatic alteration landscape

**Fig. 3:**
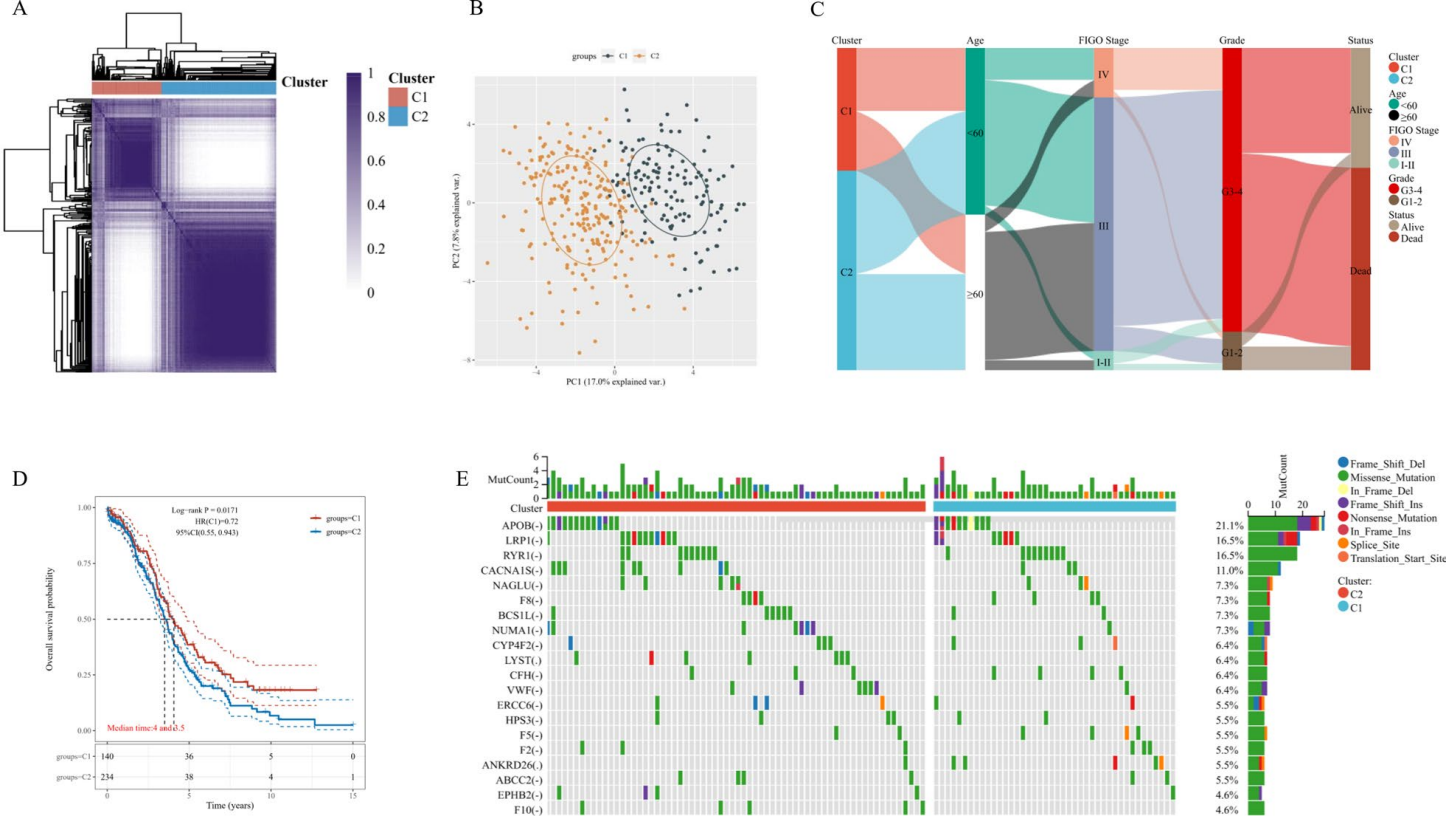
Identification of coagulation-related subtypes and somatic alteration landscape. **A** The heatmap of consensus matrices for TCGA-OV patients. To ensure clustering stability, 1000 iterations of unsupervised consensus clustering method was applied. **B** The Principal Component Analysis (PCA) analysis of coagulation subtypes in the TCGA-OV cohort. **C** The Sankey diagram for the coagulation-related subtypes and clinical features, including age, grade, FIGO stage, and survival status. **D** The Kaplan–Meier (K–M) survival curves for TCGA-OV patients, which were stratified by the coagulation-related subtypes. **E** The landscape of genomic aberrations of the genes in the two coagulation-related cluster of TCGA-OV patients. The frequency of alterations in top 20 genes were listed

The transcriptome data and corresponding clinical features of 376 patients were obtained from the TCGA-OV cohort. Based on the unsupervised clustering method, we determined two different regulation patterns, including the coagulation-related cluster 1 (n = 141) and cluster 2 (n = 235) (Fig. 3A). Through the Principal Component Analysis (PCA), patients could be divided into two remarkably different subtypes (Fig. 3B). Then, we conducted K-M survival analysis of the TCGA-OV cohort, which suggested the survival advantage of cluster 1 over cluster 2 (p-value = 0.0171) (Fig. 3C). The relationship between various clinical features and coagulation subtypes was displayed in Fig. 3D. To evaluate the genomic features of coagulation-related subtypes in OV, we visualized the Somatic cell copy number alternation (SCNA) and mutation frequency of the TCGA-OV patients. The CRGs with the highest mutation frequency are APOB (21.1%), LRP1 (16.5%), and RYR1 (16.5%) (Fig. 3E).

### The immune landscape and drug sensitivity of the coagulation subtypes

**Fig. 4:**
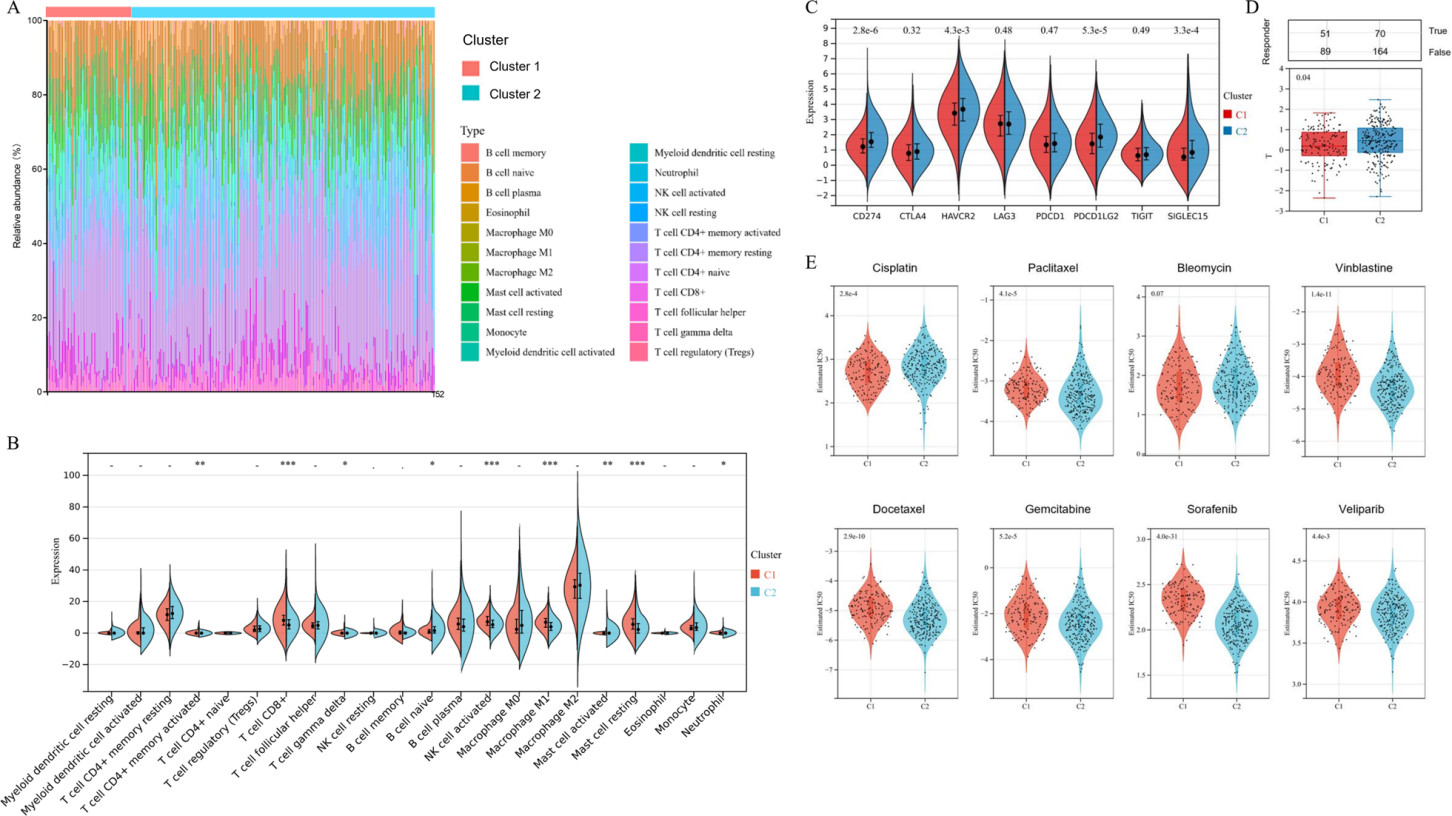
The immune landscape and drug sensitivity of the coagulation subtypes. **A** The stacked histogram showing the composition of the 22 typical immune cells infiltrating in ovarian cancer tissues, based on the CIBERSORT algorithm. **B** The violin diagram graphed the difference of 22 immune cells infiltration related to the coagulation subtypes. **C** The distribution of immune checkpoints expression between two coagulation subtypes. **D** Immune checkpoint blocking (ICB) therapy prediction for OV patients, through the Tumor Immune Dysfunction and Exclusion (TIDE) algorithm. **E** The estimated half-maximal inhibitory concentration (IC50) values of 8 common chemotherapy agents among two coagulation subtypes, based on the Genomics of Drug Sensitivity in Cancer (GDSC) database

In order to evaluated the relationship between tumor immune microenvironment and the coagulation subtypes, we analyzed the landscape of immune infiltration of 22 typical immune cells among OV patients, based on the CIBERSORT algorithm (Fig. 4A). As shown in Fig. 4B, we found that 6 out of the 22 immune cells proportions, including CD4 + memory T cells, CD8 + T cells, gamma delta T cells, activated NK cells, resting mask cells, and neutrophils were significantly up-regulated in cluster 1, while naïve B cells and active mask cells were down-regulated in cluster 1.

Subsequently, we examined the correlations between coagulation subtypes sensitivity to immunotherapy and chemotherapy. The results implied that, among main immune checkpoint molecules, only CD274, HAVCR2, PDCD1LG2, and SIGLEC15 were significantly higher in cluster 2, compared with cluster 1 (Fig. 4C, p < 0.05), which indicated that cluster 2 patients could be more likely to benefit from immunotherapies based on these typical immune checkpoints. Through the Tumor Immune Dysfunction and Exclusion (TIDE) algorithm, we found that cluster 2 patients had significantly higher TIDE score, which suggested poorer efficacy towards Immune checkpoint blocking (ICB) therapy and shorter survival after ICB therapy (Fig. 4D, P = 0.04).

Based on the Genomics of Drug Sensitivity in Cancer (GDSC) database, we also assessed chemotherapy sensitivity between two coagulation subtypes. In Fig. 4E, we estimated the half-maximal inhibitory concentration (IC50) of 8 common chemotherapy agents. The results implied that the estimated IC50 levels of Paclitaxel, Vinblastine, Docetaxel, Gemcitabine, Sorafenib, and Veliparib in cluster 1 were significantly higher, indicating that cluster 2 patients were more sensitive to these drugs. However, the IC50 levels of Cisplatin was lower in cluster 1.

### Identification of key coagulation-related genes in ovarian cancer

**Fig. 5:**
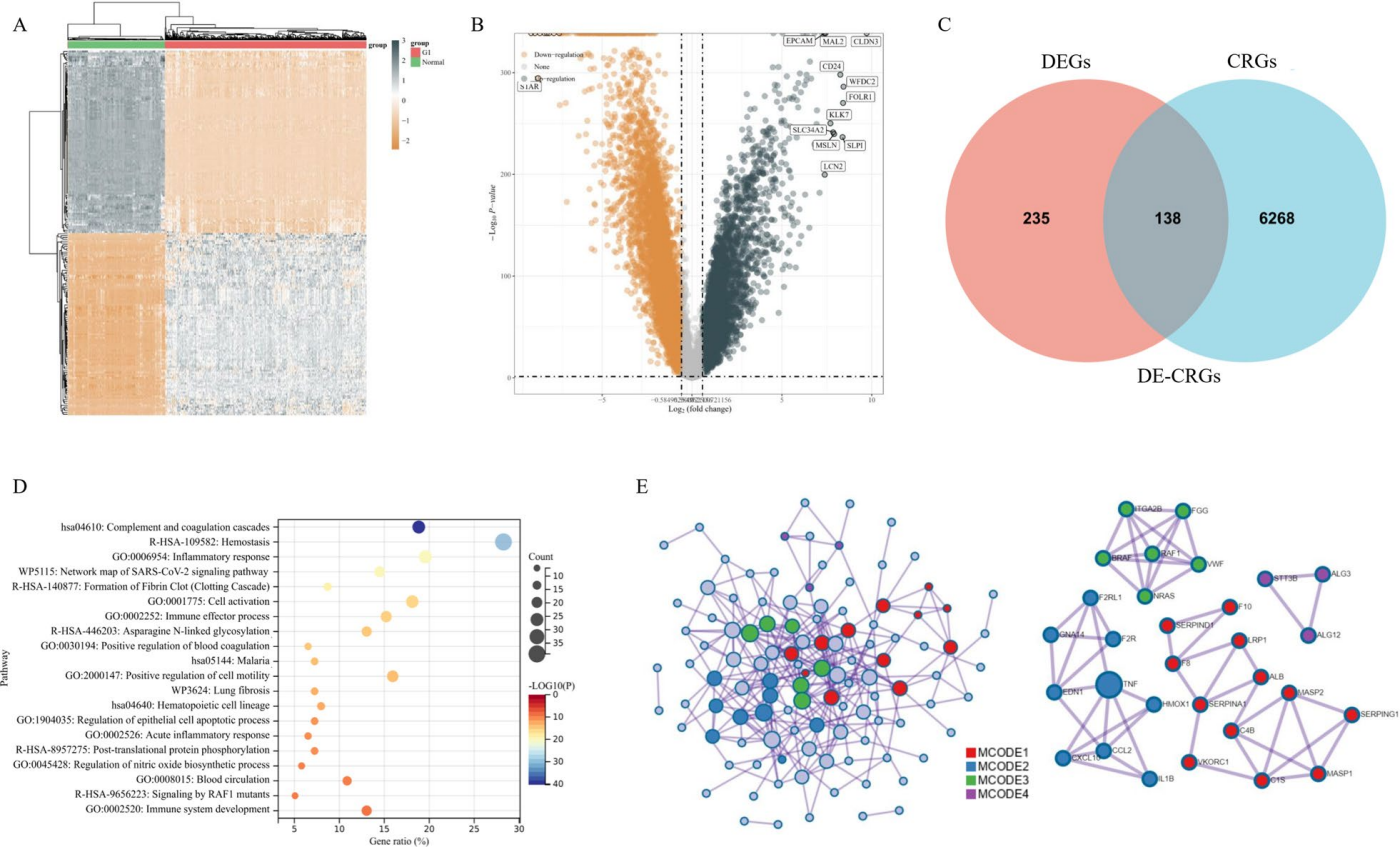
Identification of key coagulation-related genes (CRGs) in ovarian cancer (OV). **A** The heatmap diagram for differential gene expression between OV and normal tissues. **B** The volcano diagram showing the differentially-expressed genes (DEGs) between OV and normal tissues. **C** The Venn plot displaying the overlap of differentially expressed coagulation-related genes (DE-CRGs). **D** Overview of the Gene Ontology (GO) and Kyoto Encyclopedia of Genes and Genomes (KEGG) pathways enrichment analysis for the top 20 primary biological action clusters of the 138 DE-CRGs **E** The protein–protein interaction (PPI) network of all the 138 DE-CRGs

The transcriptome data from 376 patients and 180 controls was obtained from the TCGA and GTEx database, respectively. We identified 6406 differentially-expressed genes (DEGs), among which 2333 DEGs were up-regulated and 4073 DEGs were down-regulated in OV, compared with normal tissues (Fig. 5A and Fig. 5B). For further analyses, 373 CRGs were downloaded from the Genecards website (https://www.genecards.org), among which 138 CRGs were differentially expressed between OV tissues and normal controls in the Venn plot (Fig. 5C). Then, we processed pathways enrichment analysis of the 138 differentially expressed coagulation-related genes (DE-CRGs) through the Metascape website (https://metascape.org) [14] (Fig. 5D). The GO and KEGG pathways were mainly enriched in complement and coagulation cascades, inflammatory response, immune effector process, etc. In Fig. 5E, we also processed the identified DE-CRGs through the Search Tool for the Retrieval of Interacting Genes (STRING, https://string-db.org) [15], in order to graph a protein–protein interaction (PPI) network.

### Establishment and evaluation of a prognostic signature based on CRGs

**Fig. 6:**
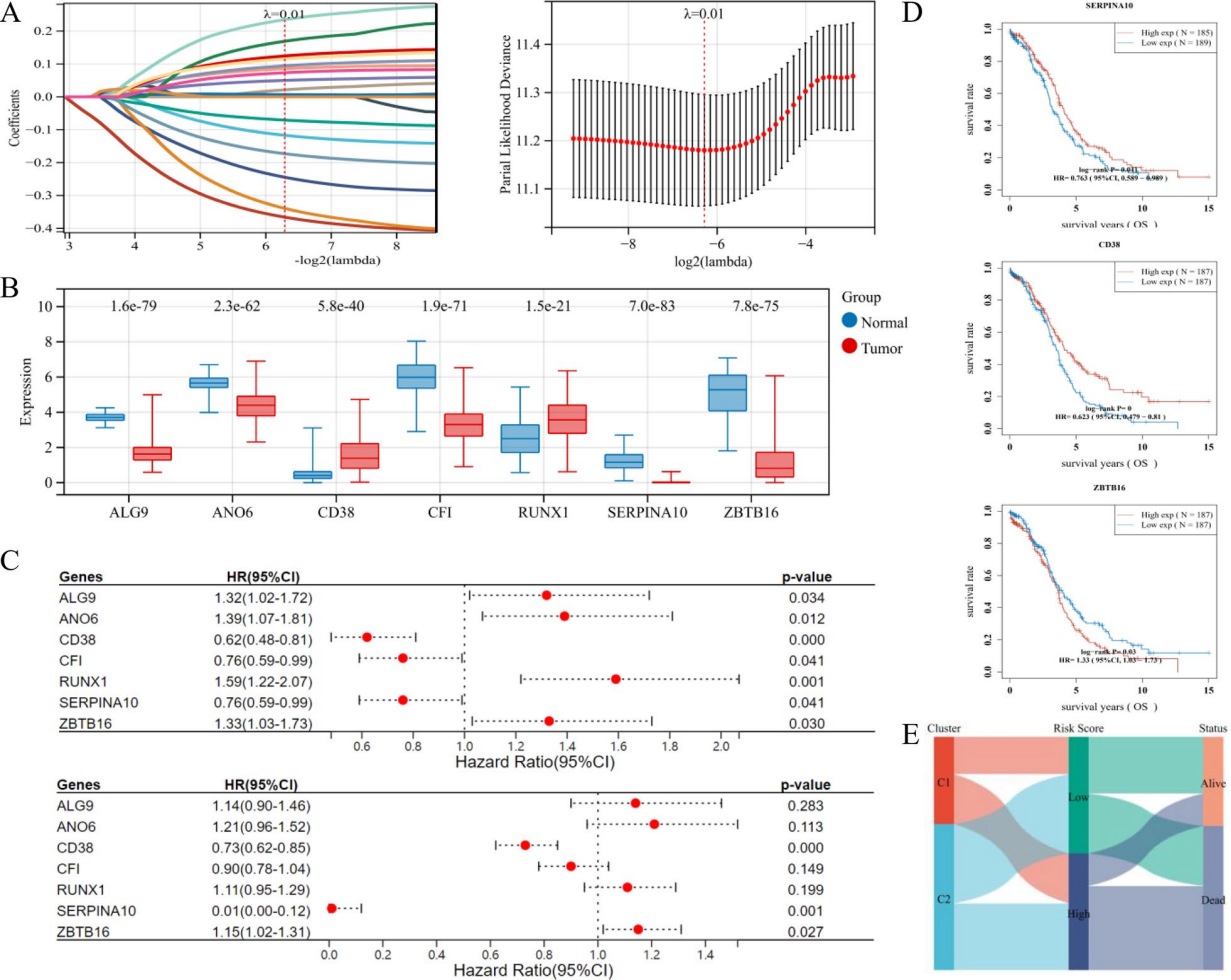
Establishment and evaluation of a prognostic signature based on coagulation-related genes (CRGs). **A** The λ selection diagram for tenfold cross-validation of LASSO regression model (left). The LASSO coefficient profiles of 7 filtered CRGs in tenfold cross-validation (right). **B** The expression distribution of the seven potential prognostic CRGs in ovarian cancer (OV) and normal tissues. **C** The forest plot of univariate (up) and multivariate Cox Regression algorithm (bottom) to distinguish prognostic CRGs. **D** The K–M survival curves of three prognostic CRGs, namely SERPINA10, CD38, and ZBTB16. **E** The Sankey diagram for the coagulation-related signature and clinical features, including age, grade, FIGO stage

In order to identify the prognostic signature, we conducted the LASSO-Cox algorithm, a common method to enhance forecast accuracy of model. From the above 138 DE-CRGs, seven prognostic genes (SERPINA10, CD38, ZBTB16, ALG9, ANO6, CFI, and RUNX1) were filtered through the LASSO algorithm (Fig. 6A). The overview of the seven potential DE-CRGs with prognosis value was listed in Table 2 [16–23]. The expressions of the seven potential prognostic CRGs in OV and normal tissues were presented in Fig. 6B.

**Table 2:** Overview of the seven potential differentially expressed coagulation-related genes (DE-CRGs) with prognosis value of ovarian cancer (OV) [16–23]

Gene	Gene name	Function in OV	Refs.
SERPINA10	Serpin Family A Member 10	SERPINA10, a member of the serpin superfamily of proteinase inhibitors related to extracellular matrix (ECM), could be a biomarker for predicting drug sensitivity and survival in platinum-based chemotherapy of OV, though the underlying mechanism is still unknown	[16]
CD38	CD38 Molecule	CD38 could predict favorable prognosis in OV, by enhancing immune infiltration and anti-tumor immunity in tumor microenvironment	[17]
ZBTB16	Zinc Finger And BTB Domain Containing 16	Unknow in OV. ZBTB16 could bind to specific DNA sequences with the C-terminal zinc fingers, so as to suppress transcription via recruiting co-repressors with the amino terminal POZ domain. ZBTB16 affects diverse signaling pathways including cell cycle, differentiation, and programmed cell death pathways in solid tumors	[18, 19]
ALG9	ALG9 Alpha-1,3-Glucosyltransferase	Unknow in OV. In acute myeloid leukemia, the mannosyl-transferase ALG9 regulates the proliferation and drug resistance in tumor cells, which could be reversed by the sponge effect of MEG3/miR-155	[20]
ANO6	Anoctamin 6	Unknow in OV. In glioma, ANO6 could promote tumor cell proliferation and invasion, by regulating the ERK signaling pathway	[21]
CFI	Complement Factor I	Unknow in OV. Complement factor I, as one of the key negative regulators of the complement system, could upregulate the expression of matrix metalloproteinase-2/-13 and promote tumor invasion in cutaneous squamous carcinoma cells	[22]
RUNX1	RUNX Family Transcription Factor 1	RUNX1, as a subunit of core-binding factors in hematopoiesis and leukemia, could regulate cisplatin-induced apoptosis in OV	[23]

**Fig. 7:**
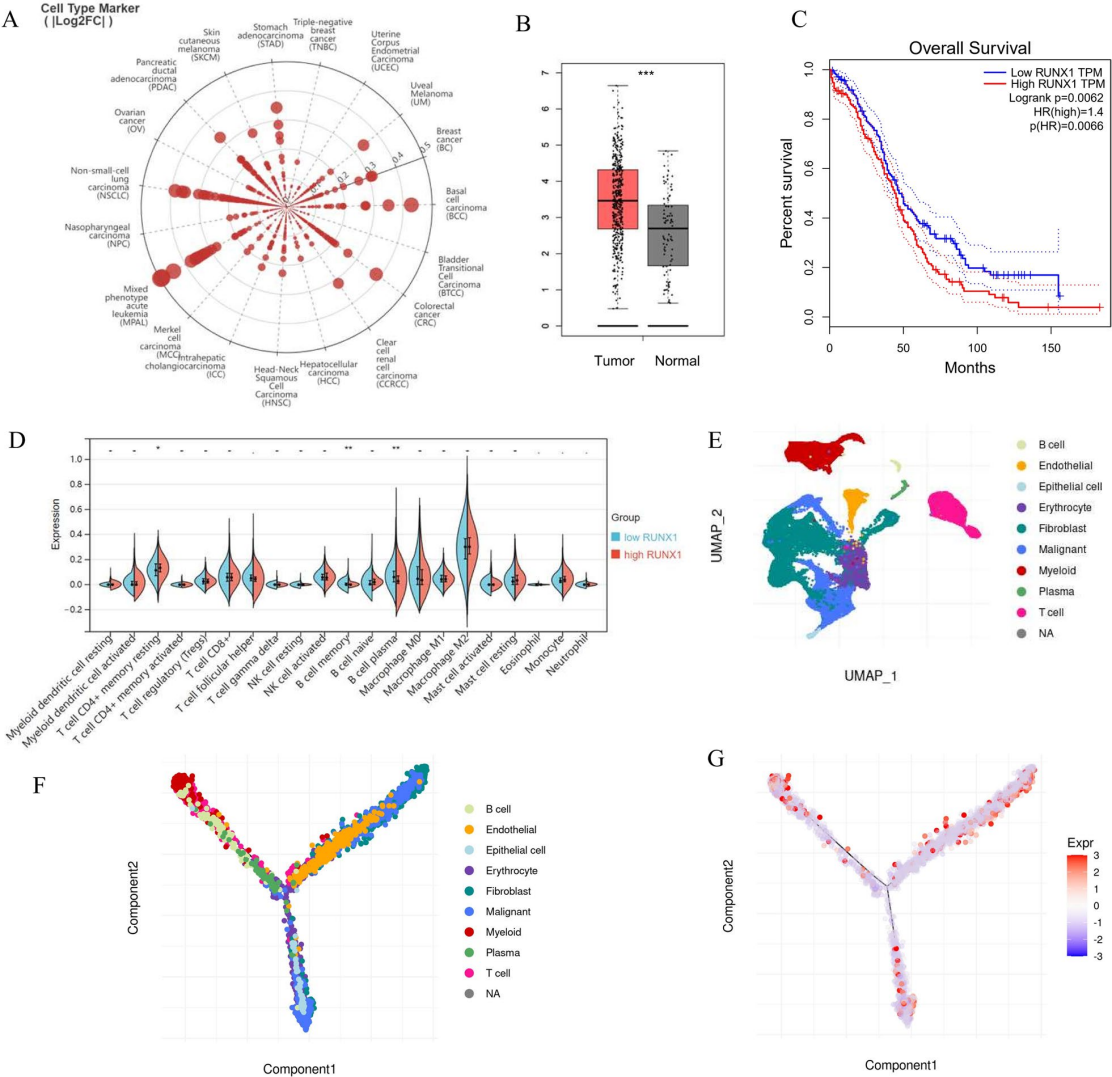
RUNX1 play a prominent role in OV prognosis and immune landscape. **A** The Radar chart represented the RUNX1 gene expression in pan-cancer. **B** The expression distribution of RUNX1 in TCGA-OV samples and GTEx normal controls were graphed. The result proved that RUNX1 was highly expressed OV tissues. **C** The Kaplan–Meier (K–M) survival curves of RUNX1 in the TCGA-OV cohort, which indicated that OV patients with higher RUNX1 suffered poor survival. **D** The Violin diagrams showed the expression of the 22 immune cells infiltration through the CIBERSORT analysis, which indicated that memory B cells and CD4 + memory T cells were significantly upregulated in patients with high RUNX1 expression, while plasma B cells were upregulated in low-RUNX1 patients. **E** The UMAP diagram showed high-quality cells from a 10 × Genomics dataset [45]. Ten typical cell types were defined by specific markers. **F** The pseudo time trajectory analysis of 10 cell types from OV tissues. **G** The pseudo time trajectory analysis of cells in OV tissues with RUNX1 expression

Among the 7 potential DE-CRGs, RUNX1 is best known for its profound and multifaceted roles in hematopoiesis at various lineage decision points [24]. At the same time, RUNX1 could contribute to the maintenance of adult stem cells in multiple epithelia, and function as a tumor suppressor in mammary epithelial cells [25, 26]. Due to its involvement in multiple cellular compartments, we deepened our analysis of RUNX1 expression and found that RUNX1 was indeed highly over-expressed in OV tissues (Fig. 7A and B), consistent with previous reports [27, 28]. Furthermore, OV patients with high RUNX1 expression have worse survival (Fig. 7C). RUNX1 over-represented tumors have significantly up-regulated memory B cells and CD4 + memory T cells, while low-RUNX1 tumors have up-regulated plasma B cells (Fig. 7D). Lastly, the pseudo time trajectory analysis of cells in OV tissues with RUNX1 expression revealed that RUNX1 is over-expressed in myeloid cells and malignant OV cells, providing further support for a positive correlation between high RUNX1 expression and OV malignancy (Fig. 7E–G). As RUNX1 has two promoters: P1 (most active in hematopoietic lineages) and P2 (active in epithelial cells) [29, 30], we examined the expression of various RUNX1 isoforms in OV (Additional file 1: Figure S1A). The results revealed that the ENST00000344691.8 isoform, one of the P2 transcripts, had the highest expression in OV tissues, suggesting a significant contribution from epithelial cells (Additional file 1: Figure S1B). Nevertheless, there was no difference between the overall expression of P1 and P2 transcripts, reflecting the multiple cellular sources of RUNX1 transcripts within OV tumors (Additional file 1: Figure S1C).

Then, we used both univariate and multivariate Cox Regression algorithm to distinguish prognostic genes, namely SERPINA10, CD38, and ZBTB16 (Fig. 6C). Ultimately, the prognostic signature was established as following: Riskscore = −3.458*SERPINA10–0.269*CD38 + 0.159*ZBTB16. The K-M survival curves indicated that OV patients with up-regulated SERPINA10 and CD38 had better OS, while those with up-regulated ZBTB16 suffered worse OS (Fig. 6D). We also graphed the distribution of each patient, according to different clinical variables and the risk groups classified by the coagulation signature (Fig. 6E).

**Fig. 8:**
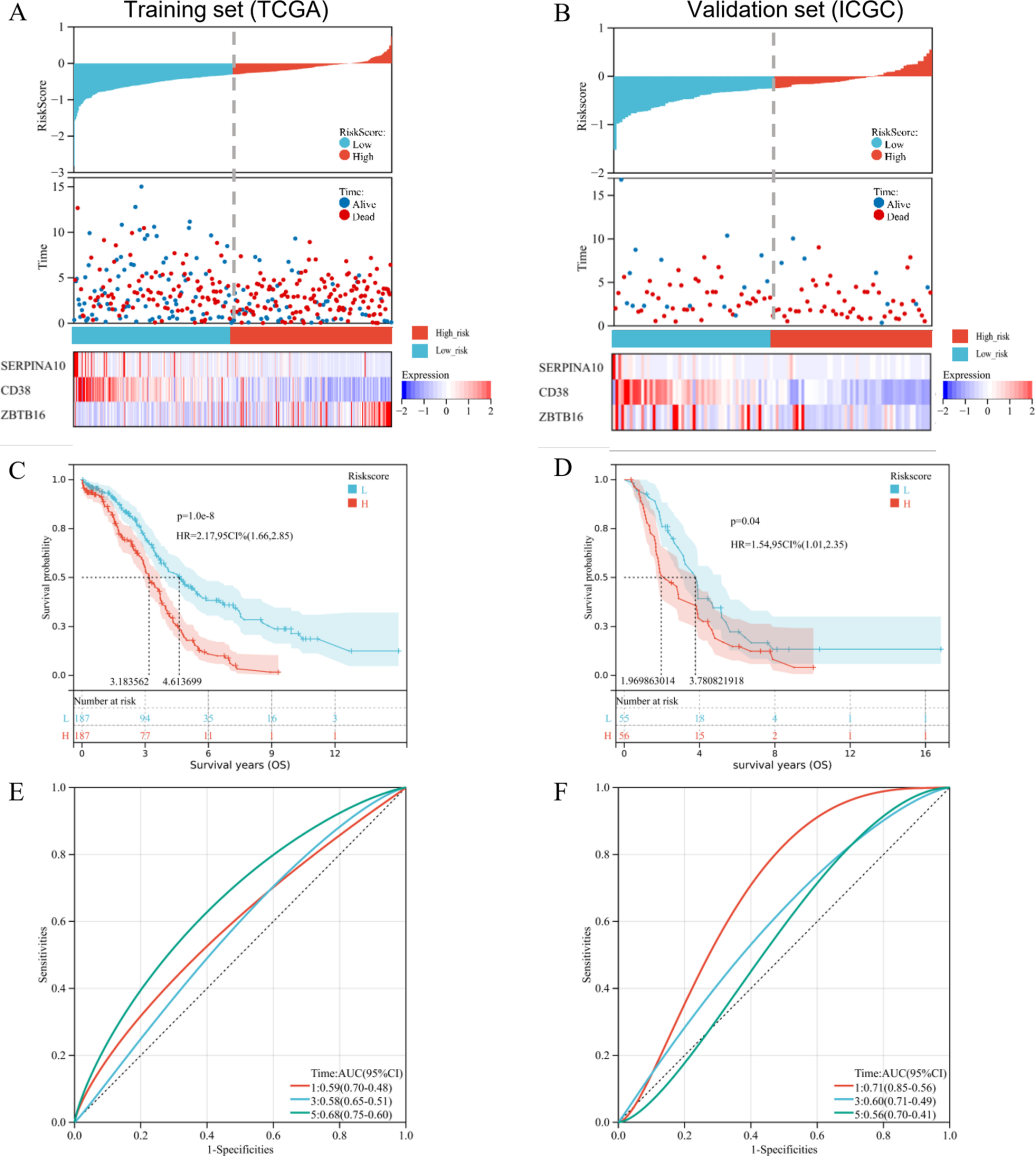
Survival evaluation of the signature based on coagulation-related genes (CRGs) in ovarian cancer (OV). The distribution of riskscore, survival status, and survival time for each patient in both **A** training set (TCGA-OV, n = 376) and **B** validation set (ICGC-OV, n = 111). The histogram represented patients stratified into two groups according to the median cut-off value (top). The scatter plot graphed riskscores corresponding to survival time and status (middle). The heatmap showed gene expression of the three CRGs (bottom). The Kaplan–Meier survival curves for overall survival (OS) in high-risk and low-risk groups of the **C** training set and **D** validation set. The time-dependent Receiver Operating Characteristic (ROC) analysis for 1-year, 3-year, and 5-year OS prediction in **E** training set and **F** validation set

Based on the above formula, we calculated the riskscore of every patient in both training set (TCGA-OV, n = 376) and validation set (ICGC-OV, n = 111). Then, we stratified them into two groups according to the median cut-off value (Fig. 8A and B), top). In high-risk and low-risk groups, we also evaluated the distribution of survival status of all patients and expression profiles of the three prognostic genes (Fig. 8A and B), middle and bottom). Most of death cases were distributed among the high-risk group, while SERPINA10 and CD38 were highly expressed in the low-risk group. The K-M survival curves illustrated that high-risk patients suffered worse OS than low-risk patients in training set (p < 0.001, Fig. 8C) and validation set (p = 0.040, Fig. 8D). Additionally, refer to the time-dependent ROC analysis, the coagulation-associated signature had promising AUC values for 1-year, 3-year, and 5-year OS prediction in both training and validation sets (Fig. 8E and 8F). These findings demonstrated the reliable prognostic ability of the defined signature.

### Construction and validation of the coagulation-associated nomogram

**Fig. 9:**
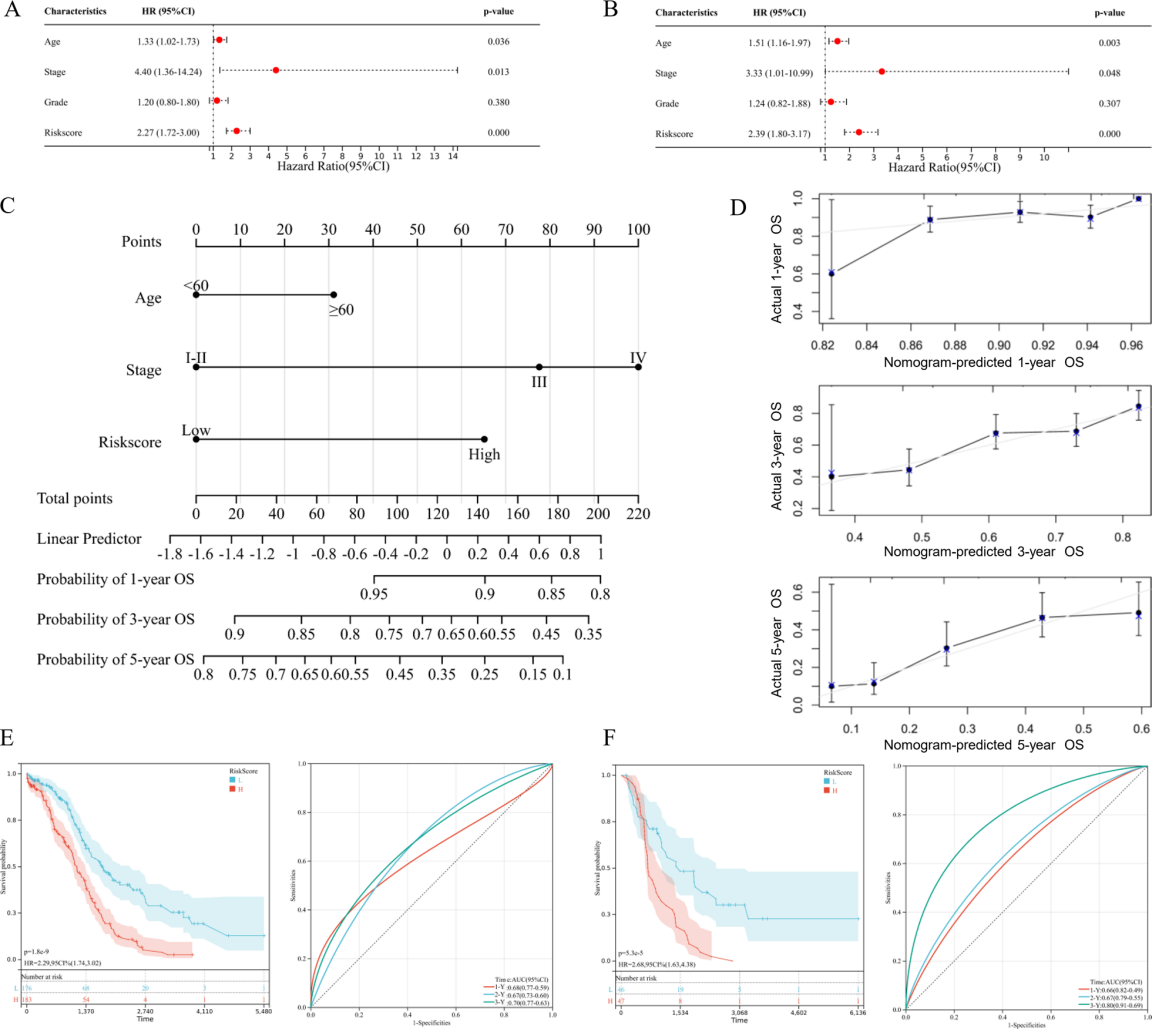
Construction and validation of the coagulation-associated nomogram for ovarian cancer (OV). The forest plot for (**A**) univariate and (**B**) multivariate Cox Regression analysis of OV survival, containing the coagulation-associated signature and clinical features. **C** The quantitative nomogram for 1-year, 3-year, and 5-year overall survival (OS) prediction in the TCGA cohort. **D** The calibration plots for consistency between predicted and observed 1-year, 3-year, and 5-year survival. The Kaplan–Meier (K–M) survival curves (left) and time-dependent ROC curves (right) for the **E** TCGA cohort and **F** ICGC cohort, which were stratified by the nomogram score

As the CRGs were significantly correlated with poor survival in OV, we performed both univariate (Fig. 9A) and multivariate (Fig. 9B) Cox Regression analyses to determine all the independent prognostic factors for OV. The results confirmed that riskscore (p = 0.000), FIGO stage (p = 0.048) and age (p = 0.003) were prognostic factors for OS. Based on the filtered factor, we constructed a quantitative nomogram for OS prediction, with the C-index of 0.6761(95% CI 0.6331–0.7191) (Fig. 9C). Calibration plots indicated ideal consistency between predicted and observed 1-year, 3-year, and 5-year survival (Fig. 9D). Furthermore, through K-M curve analysis and time-dependent ROC analysis, we validated the optimum performance of the nomogram in both TCGA cohort (Fig. 9E) and ICGC cohort (Fig. 9F).

### Pan-cancer analysis of the coagulation-associated signature

**Fig. 10:**
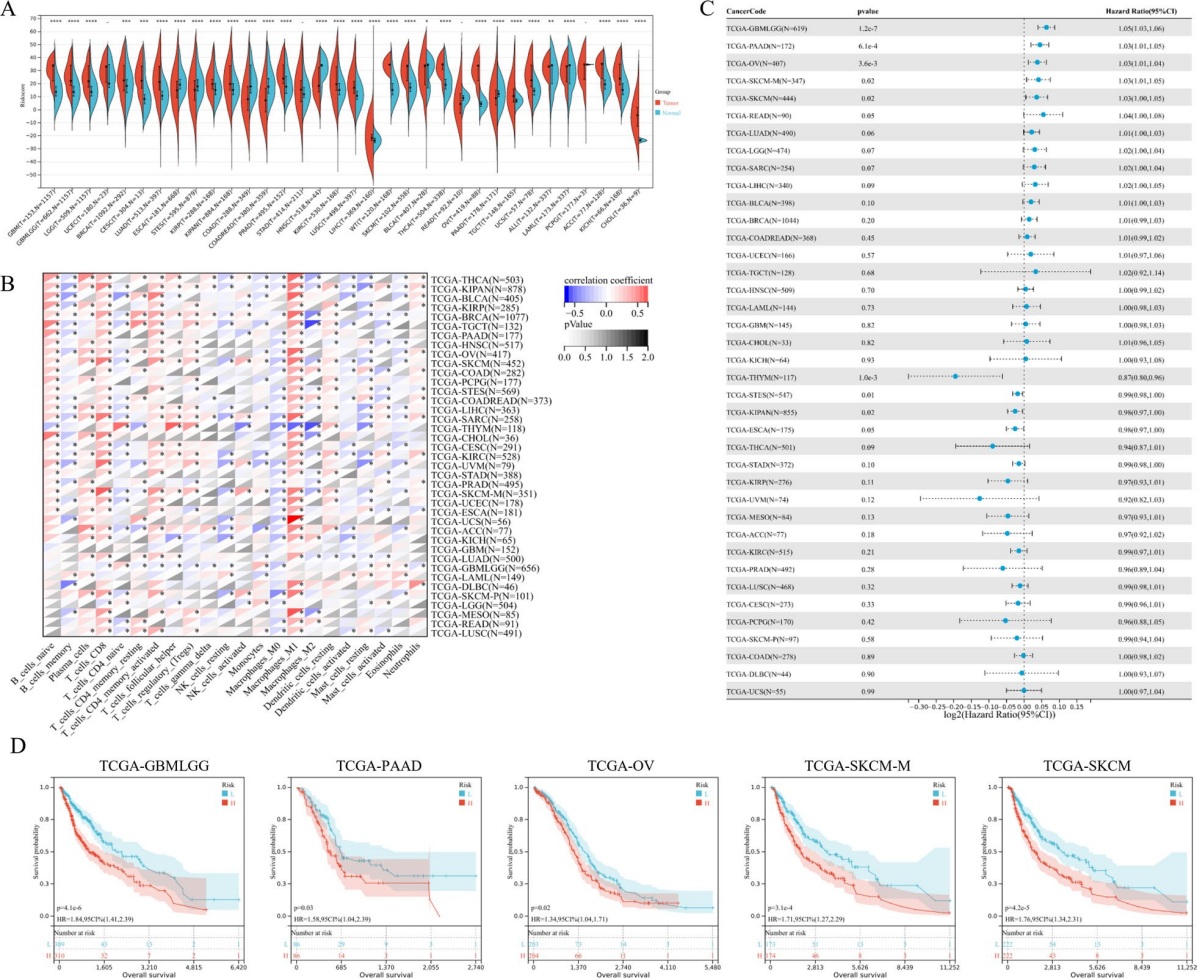
Pan-cancer analysis of the coagulation-associated signature. **A** The violin plots presented the riskscore level of tumor tissues and normal controls in 34 tumors. **B** The relationship between riskscore and immune cell infiltration level in pan-cancer, which was analyzed through the CIBERSORT algorithm. **C** The forest plot of the Cox Regression algorithm to distinguish the prognostic value of the coagulation-associated signature in pan-cancer. **D** The Kaplan–Meier (K–M) survival curves for glioma (GBMLGG), pancreatic adenocarcinoma (PAAD), ovarian serous cystadenocarcinoma (OV), skin cutaneous melanoma (SKCM), and SKCM-M in the TCGA cohorts, which were stratified by the coagulation-associated signature

In order to determine application of the coagulation-associated signature in cancers, we performed pan-cancer analysis on 34 tumors in the TCGA cohorts. Firstly, we compared the riskscore level of tumor tissues and normal controls, among which almost all cancers had different riskscore level, except for uterine corpus endometrial carcinoma (UCEC), stomach adenocarcinoma (STAD), rectum adenocarcinoma (READ), and pheochromocytoma and paraganglioma (PCPG), while adrenocortical carcinoma (ACC) ranked the highest riskscore (Fig. 10A). Furthermore, we explored the relationship between immune cell infiltration and the coagulation-associated signature in pan-cancer (Fig. 10B). The results suggested that CD8 + T cells and Macrophage M1 cells were positively related to the signature in pan-cancer, while Macrophage M0 cells were inversely related. The prognostic value of the coagulation-associated signature was also evaluated in pan-cancer cohorts through the Cox Regression algorithm (Fig. 10C). Through K–M curve analysis, we validated the optimum performance of the signature (p < 0.05) in glioma (GBMLGG), pancreatic adenocarcinoma (PAAD), ovarian serous cystadenocarcinoma (OV), skin cutaneous melanoma (SKCM), and SKCM-M cohorts (Fig. 10D).

### Aberrant upregulation of ZBTB16 in OV was related with metastasis and poor prognosis

We involved 36 OV individuals in our institution, which were followed up for the media time of 37.88 (31.48–42.72) months. The clinical characteristics of involved OV patients were showed in Additional file 3: Table S3. We measured the RNA expression of SERPINA10, CD38, and ZBTB16 in the OV tissues through qRT-PCR analysis, which revealed that lower SERPINA10 and CD38 expressions were found in patients suffered poor prognosis, while higher ZBTB16 expression was found among them (p < 0.05, Additional file 2: Figure S2A–C). We also conducted the Western Blotting, which further proved that protein expression of ZBTB16 significantly increased in metastatic lesions, while protein expression of SERPINA10 and CD38 expression decreased in metastatic samples (Additional file 2: Figure S2D). Stepwise, we conducted both univariate (Additional file 2: Figure S2E) and multivariate Cox regression analyses (Additional file 2: Figure S2F) for prognostic clinical features. The results indicated that SERPINA10, CD38, and ZBTB16 (p = 0.046, 0.040, and 0.008, respectively) were prognostic factors, in addition to the FIGO stage (p-value = 0.008). In Additional file 2: Figure S2G-I, we concluded that patients with higher ZBTB16 expression suffered worse OS, while those with higher SERPINA10 and CD38 expression had better prognosis (p < 0.05), through the K–M survival curves. The findings were consisted with the results of bioinformatics analysis.

**Fig. 11:**
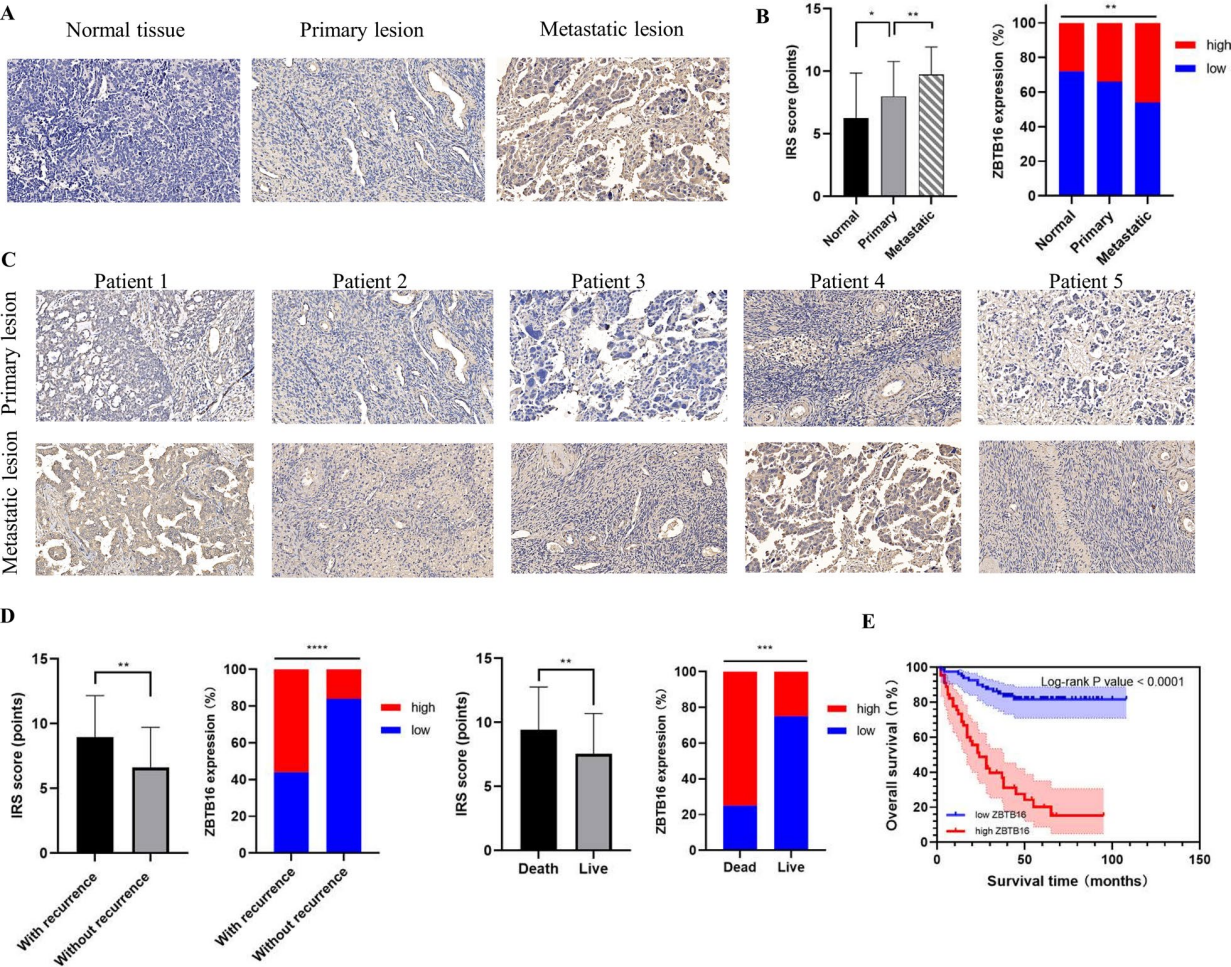
ZBTB16 expression is up-regulated in OV and related to poor prognosis. **A** The representative immunohistochemistry (IHC) staining images of ZBTB16 expression of various specimens (including primary OV lesions, metastatic lesions, and normal controls) were listed. Original magnification × 200. **B** Compared with primary OC lesions and normal controls, metastatic lesions had higher ZBTB16 expression, measured through IRS score. **C** The IHC staining images of primary and metastatic tumor lesions from 5 representative OV patients. Original magnification × 200. **D** ZBTB16 expression was increased in patients who suffered recurrence or death, measured through IHC staining analysis of the tissue microarrays. **E** The Kaplan–Meier survival curves for the overall survival (OS, bottom) of 125 OV patients were shown, which reveal that the upregulation of ZBTB16 correlates with poor survival

The IHC analysis of tissue microarrays demonstrated that ZBTB16 expression staining was mainly located at cytosol of tumor cells (Fig. 11A). Moreover, metastatic lesions had significantly higher ZBTB16 expression (IRS score = 9.73 ± 2.22), compared with primary OV lesions (IRS score = 8.54 ± 2.52) and normal ovary tissues (IRS score = 6.11 ± 3.72) (Fig. 11B). The IHC staining images of primary and metastatic tumor lesions from 5 representative OV patients were shown in Fig. 11C. Through IHC staining analysis of the tissue microarrays based on 125 OC cases, we found that ZBTB16 expression was increased among patients who suffered recurrence or death, compared with survivors (Fig. 11D). The association between ZBTB16 expression and clinicopathological characteristics of all OV patients was listed in Table 3, only with significant differences among various ZBTB16 expression groups refer to the FIGO stage (p-value = 0.014).

**Table 3:** The correlation between ZBTB16 expression and clinicopathological characteristics of 125 OV patients

Characteristic	No. of patients	ZBTB16 expression		p-value
		Low (IRS score < 8)	High (IRS score ≥ 8)	
Age (n,%)				0.590
< 55 years	56 (44.8%)	33 (26.4%)	23 (18.4%)	−
≥ 55 years	69 (55.2%)	37 (29.6%)	32 (25.6%)	−
FIGO stage (n,%)				0.014
I–II	45 (36.0%)	32 (25.6%)	13 (10.4%)	−
III–IV	80 (64.0%)	38 (30.4%)	42 (33.6%)	−
Pathology stage (n,%)				0.587
I–II	54 (43.2%)	32 (25.6%)	22 (17.6%)	−
III	71 (56.8%)	38 (30.4%)	33 (26.4%)	−
Histology type (n,%)				0.068
Serous	78 (62.4%)	47 (37.6%)	31 (24.8%)	−
Mucous	11 (8.8%)	8 (6.4%)	3 (2.4%)	−
Endometrioid	14 (11.2%)	8 (6.4%)	6 (4.8%)	−
Other types	22 (17.6%)	7 (5.6%)	15 (12.0%)	−
Tumor diameter (n,%)				0.473
< 10 cm	62 (49.6%)	37 (29.6%)	25 (20.0%)	−
≥ 10 cm	63(50.4%)	33 (26.4%)	30 (24.0%)	−
Serum CA125 (n,%)				0.221
< 35 U/ml	20 (16.0%)	14 (11.2%)	6 (4.8%)	−
≥ 35 U/ml	105 (84.0%)	56 (44.8%)	49 (39.2%)	−

*FIGO stage* Federation of International of Gynecologists and Obstetricians stage

The median OS and PFS for involved OV patients were 34 (range 14–52) months and 59 (range 25–89) months, respectively. The K–M survival analysis revealed that ZBTB16 expression was significantly related to OS (p < 0.001, Fig. 11E) among OC patients. Then, we performed both univariate and multivariate analyses to determine independent prognostic factors (Table 4). The results indicated that FIGO stage (HR 2.624; 95% CI 1.050–6.557; p = 0.039) and ZBTB16 expression (HR 5.012; 95% CI 2.513–9.994; p = 0.001) were significantly associated with OV prognosis. Collectively, ZBTB16, one of the key CRGs, was significantly associated with OC metastasis and poor prognosis.

**Table 4:** Univariate and multivariate analysis of OV prognostic factors

Characteristic	Univariate analysis		Multivariate analysis	
	HR (95% CI)	P-value	HR (95% CI)	P-value
Age				
< 55 years	Reference	−	Reference	−
≥ 55 years	1.052(0.595–1.862)	0.861	1.014(0.556–1.842)	0.97
FIGO stage				
I–II	Reference	−	Reference	−
III–IV	4.238(1.897–9.471)	0.001	2.624(1.050–6.557)	0.039
Pathology grade				
I–II	Reference	−	Reference	−
III	0.727(0.413–1.282)	0.271	0.673(0.348–1.302)	0.239
Histology type		0.850		0.934
Serous	Reference	−	Reference	−
Mucous	0.591(0.181–1.932)	0.384	1.498(0.311–7.220)	0.614
Endometrioid	0.875(0.341–2.245)	0.781	1.305(0.475–3.585)	0.606
Other types	0.977(0.450–2.122)	0.953	1.021(0.409–2.546)	0.965
Tumor diameter				
< 10 cm	Reference	−	Reference	−
≥ 10 cm	1.253(0.710–2.211)	0.437	1.364(0.749–2.482)	0.310
Serum CA125				
< 35 U/ml	Reference	−	Reference	−
≥ 35 U/ml	2.680(0.960–7.486)	0.061	2.093(0.511–8.572)	0.305
ZBTB16 expression				
Low (IRS score < 8)	Reference	−	Reference	−
High (IRS score ≥ 8)	5.815(3.009–11.236)	0.001	5.012(2.513–9.994)	0.001

*FIGO stage* Federation of International of Gynecologists and Obstetricians stage

## Discussion

OV is the most lethal gynecological cancers, with increasing incidence and poor prognosis worldwide [1]. Recently, emerging studies has reported the interactions between coagulation and malignant tumor progression in OV [31]. The production and activation of procoagulant factors, including tissue factor (TF), microparticles (MPs), proangiogenic factors, and cytokines, could promote tumorigenesis and cancer development, which could finally result in a chronic hypercoagulable state and affect immune microenvironment [32]. So far, the mechanism of the relationship between coagulation pathway and cancer prognosis or immune microenvironment has remained largely unknown [33]. Hence, we aimed to clarify the role of coagulation pathway in prognosis, immune microenvironment, and therapeutic response in OV.

In our previous research, we estimated the association between coagulation and OV, by demonstrating the coagulation indexes as prognostic factors for OV patients [11]. Stepwise, in this study, we further evaluated the prognostic value of more coagulation variables, including APTT, PT, TT, fibrinogen, and INR. For the first time, we defined the F-INR score, based on two filtered prognostic coagulation indexes, namely fibrinogen and INR. Previous studies indicated that fibrinogen, the coagulation factor I transformed from fibrin by activated thrombin, could lead to clot formation in the coagulation pathway [34]. Meanwhile, the INR system, which was applied to standardize PT, could evaluate the “extrinsic coagulation pathway” in patients [35]. Accordingly, both indexes were deemed as reliable indicators for in vivo coagulation status, while hypercoagulation could lead to poor prognosis [7]. However, there is still an ongoing blank over the underlying mechanisms of relationship between coagulation factors and OV prognosis, thus further in-depth insights are needed.

Moreover, in this research, we divided OV patients into two remarkably different subtypes based on CRGs expression. The K-M survival analysis showed the survival advantage of cluster 1 over cluster 2 (p-value = 0.0171). Clinical characteristics analysis suggested that there were more advanced OV cases in cluster 2, which might explain the poor prognosis in this cluster. Considering the potential relationship between coagulation and immune activation, we investigated the landscape of immune infiltration between two coagulation-related clusters. In our study, CD4 + memory T cells, CD8 + T cells, gamma delta T cells, activated NK cells, resting mask cells, and neutrophils were significantly infiltrated in cluster 1 more than cluster 2, while naïve B cells and active mask cells infiltrated in cluster 2. The relationship between tumor cells and immune microenvironment is extremely complex, while different immune cells have different roles. Fridlender and colleagues reported that neutrophils are a vital part of the TME, which could be polarized into the anti-tumor (N1) or pro-tumor (N2) phenotypes [36]. Our previous study also demonstrated that increased neutrophil was a poor prognostic biomarker for OV patients [37]. A recent study claimed that CD8 + T cells were cytotoxic cells that could induce anti-tumor response through producing interferon-γ (IFN-γ) [38], which is consistent with our findings. Accordingly, compared with cluster 2, cluster 1 was significantly associated with immune-activation, which might lead to a better prognosis.

Nowadays, immunotherapy, including immune checkpoint blockade, cancer vaccines, and adoptive cell therapy has attracted great interest with improved understanding of the molecular basis of immune regulation of cancer cells [39]. For instance, our research team has developed the “mini DCs”, a nano-vaccine inherited the ability of T cells’ stimulation and antigen presentation from DCs, which could exhibit superior prophylactic and therapeutic efficacy against cancer in the mouse model of OV [40]. However, partly due to the immune suppressive networks within the OV tumor microenvironment, only a few OV patients could benefit from immunotherapy [41]. Therefore, a major direction is to investigate effective biomarkers that could predict responsiveness to various immunotherapies, in order to allow precise treatment selection. In our study, we found that the CRGs was correlated with immune checkpoint molecules and tumor immune landscape, which indirectly indicated that coagulation might play a vital role in forecasting immunotherapy response. Especially, the CRGs was found to be an effective predictor for immunotherapies based on immune checkpoints including CD274, HAVCR2, PDCD1LG2, and SIGLEC15, though the underlying mechanisms still need further elucidation.

After screening based on the LASSO-COX algorithms, we identified 3 key CRGs (SERPINA10, CD38, and ZBTB16). SERPINA10, also known as Protein Z-dependent proteinase inhibitor (ZPI), could inhibit activated factor X (FXa) in the coagulation process associated with protein Z (PZ), calcium ions, and cephalin [42]. Guo and colleagues reported that SERPINA10 was a biomarker for predicting platinum sensitivity and survival benefits for OV, though the underlying mechanism is still unknown [16]. CD38, a multifunctional transmembrane glycoprotein with ADP-ribosyl cyclase activity, is known to be expressed on platelets [43]. CD38 plays an essential role in thrombin-induced procoagulant activity of platelets and hemostasis through catalyzing the formation of intracellular Ca(2 +) messengers [44]. Consist with our findings, Zhu and colleagues concluded that CD38 could predict favorable prognosis in OV, by enhancing immune infiltration and anti-tumor immunity in tumor microenvironment [17]. Among 3 selected CRGs, ZBTB16 was the only key genes that was upregulated in OV patients with poor prognosis. Recent studies have demonstrated ZBTB16 could bind to specific DNA sequences with the C-terminal zinc fingers, which could suppress transcription through recruiting co-repressors with amino terminal POZ domain [18]. Moreover, Brunner and colleagues reported that ZBTB16 could affect diverse signaling pathways including cell cycle, differentiation, and programmed cell death pathways in solid tumors, though still unknown in OV [19]. In our study, we reported that the aberrant upregulation of ZBTB16 in OV was related with metastasis and poor prognosis through bioinformation analysis at the very first time. We also validated the findings at mRNA and protein level, through tissue microarrays analysis, Western Blot, and qRT-PCR.

However, there were also some limitations in our study. Firstly, the number of cases in the TCGA-OV cohort is still limited. Hence more large-scale datasets are needed to verify the findings. Moreover, although the interaction between immune microenvironment and coagulation pathways were found in the OV patients, the underlying biological mechanisms were still unclear. So, further functional and mechanistic experiments are needed to verify the roles of the coagulation pathways in OV.

## Conclusion

Nonetheless, our study demonstrated that coagulation was associated with immune infiltration and prognosis in OV. We constructed a novel coagulation-related 3-gene signature, including SERPINA10, CD38, and ZBTB16, which could provide a robust prognostic tool and facilitate clinical guidance for OV patients. Based on the coagulation-related signature and clinical features, we developed a nomogram model for predicting the survival of OV patients within 1–5 years. To validate our findings, we also demonstrated that the aberrant upregulation of CRGs in OV tissues was related with metastasis and poor prognosis. In sum, our systematic study of CRGs revealed the vital role of coagulation in OV, providing a new perspective for individual treatment.

### Supplementary Information

**Additional file 1: Figure S1.** The overview of RUNX1 isoforms in ovarian cancer (OV). **A** The structure of different RUNX1 isoforms. **B** The expression of various RUNX1 isoforms in OV, among which the ENST00000344691.8 isoform had the highest expression. **C** The expression levels of P1 and P2 *RUNX1* transcripts in OV.

**Additional file 2: Figure S2.** The coagulation-related genes could predict prognosis for ovarian cancer (OV) patients. The gene expression of (**A**) CD38, (**B**) SERPINA10, and (**C**) ZBTB16 in OV tissues, which was evaluated through the qRT-PCR analysis. (**D**) The Western blotting analysis showed the protein expression of CD38, SERPINA10, and ZBTB16 in primary and metastatic lesions from representative OV patients. The (**E**) univariate and (**F**) multivariate Cox regression analysis for OV patient survival, based on clinical features and three coagulation-related genes. The Kaplan–Meier (**K**–**M**) curves for OV patients, which were stratified by the expression of (**G**) CD38, (**H**) SERPINA10, and (**I**) ZBTB16.

**Additional file 3: Table S3.** The baseline characteristics of ovarian cancer (OV) patients.

## Data Availability

The data that support the findings of this research are available from the corresponding author upon reasonable requests.
