# Supplementary material for: Clinical significance and immune infiltration analyses of a novel coagulation-related signature in ovarian cancer
Source: Cancer Cell Int. 2023 Oct 6;23:232. doi: 10.1186/s12935-023-03040-3 (PMC10559580; doi:10.1186/s12935-023-03040-3)

**Supplement figure 1: The overview of RUNX1 isoforms in ovarian cancer (OV).** (A) The structure of different RUNX1 isoforms. (B) The expression of various RUNX1 isoforms in OV, among which the ENST00000344691.8 isoform had the highest expression. (C) The expression levels of P1 and P2 *RUNX1* transcripts in OV.


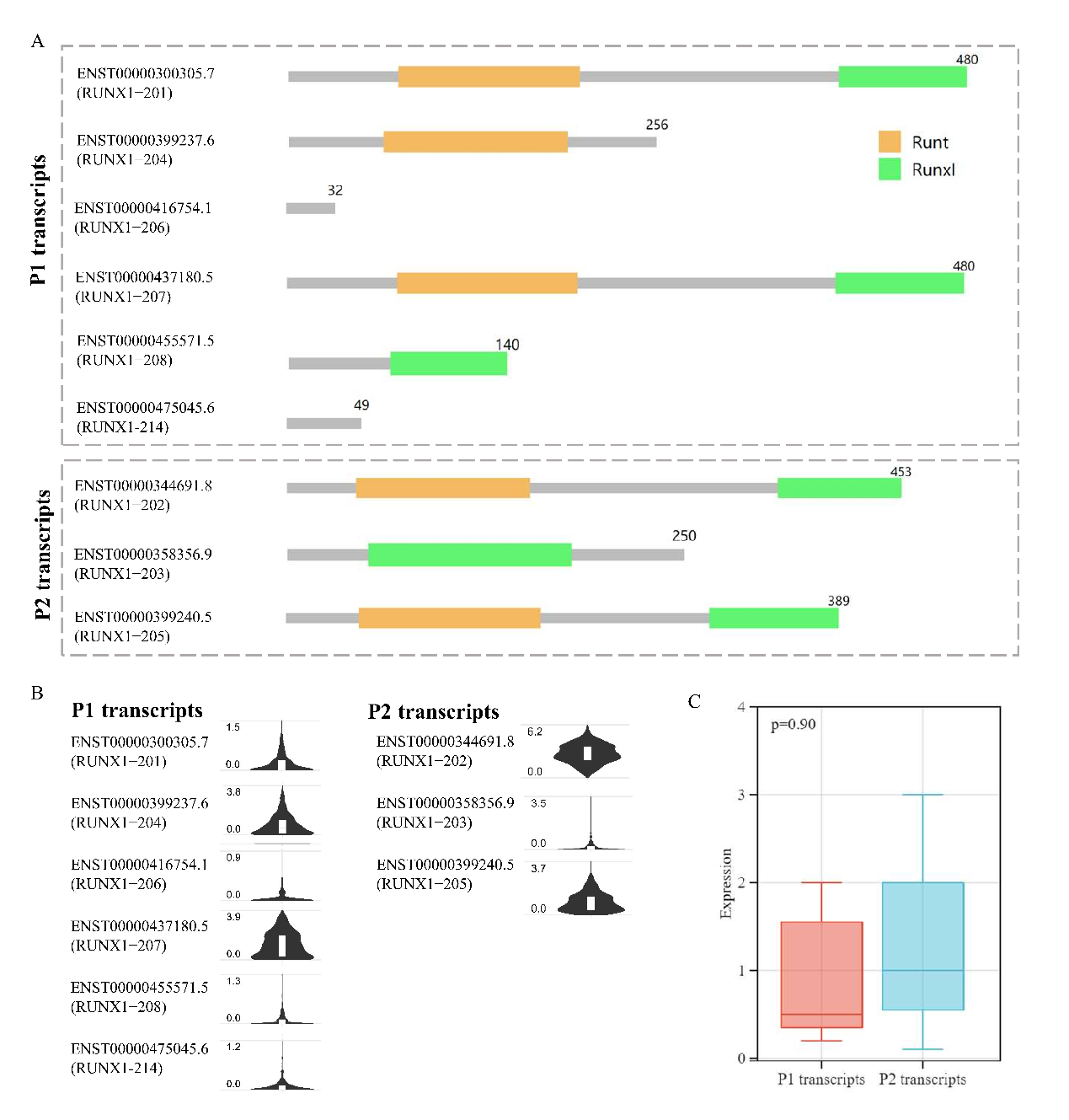

Supplement: Supplementary file 1 — Additional file 1: Figure S1. The overview of RUNX1 isoforms in ovarian cancer (OV). A The structure of different RUNX1 isoforms. B The expression of various RUNX1 isoforms in OV, among which the ENST00000344691.8 isoform had the highest expression. C The expression levels of P1 and P2 RUNX1 transcripts in OV. [file 12935_2023_3040_MOESM1_ESM.docx]
