# Supplementary material for: Clinical significance and immune infiltration analyses of a novel coagulation-related signature in ovarian cancer
Source: Cancer Cell Int. 2023 Oct 6;23:232. doi: 10.1186/s12935-023-03040-3 (PMC10559580; doi:10.1186/s12935-023-03040-3)

**Supplement figure 2. The coagulation-related genes could predict prognosis for ovarian cancer (OV) patients.** The gene expression of (A) CD38, (B) SERPINA10, and (C) ZBTB16 in OV tissues, which was evaluated through the qRT-PCR analysis. (D) The Western blotting analysis showed the protein expression of CD38, SERPINA10, and ZBTB16 in primary and metastatic lesions from representative OV patients. The (E) univariate and (F) multivariate Cox regression analysis for OV patient survival, based on clinical features and three coagulation-related genes. The Kaplan-Meier (K-M) curves for OV patients, which were stratified by the expression of (G) CD38, (H) SERPINA10, and (I) ZBTB16.


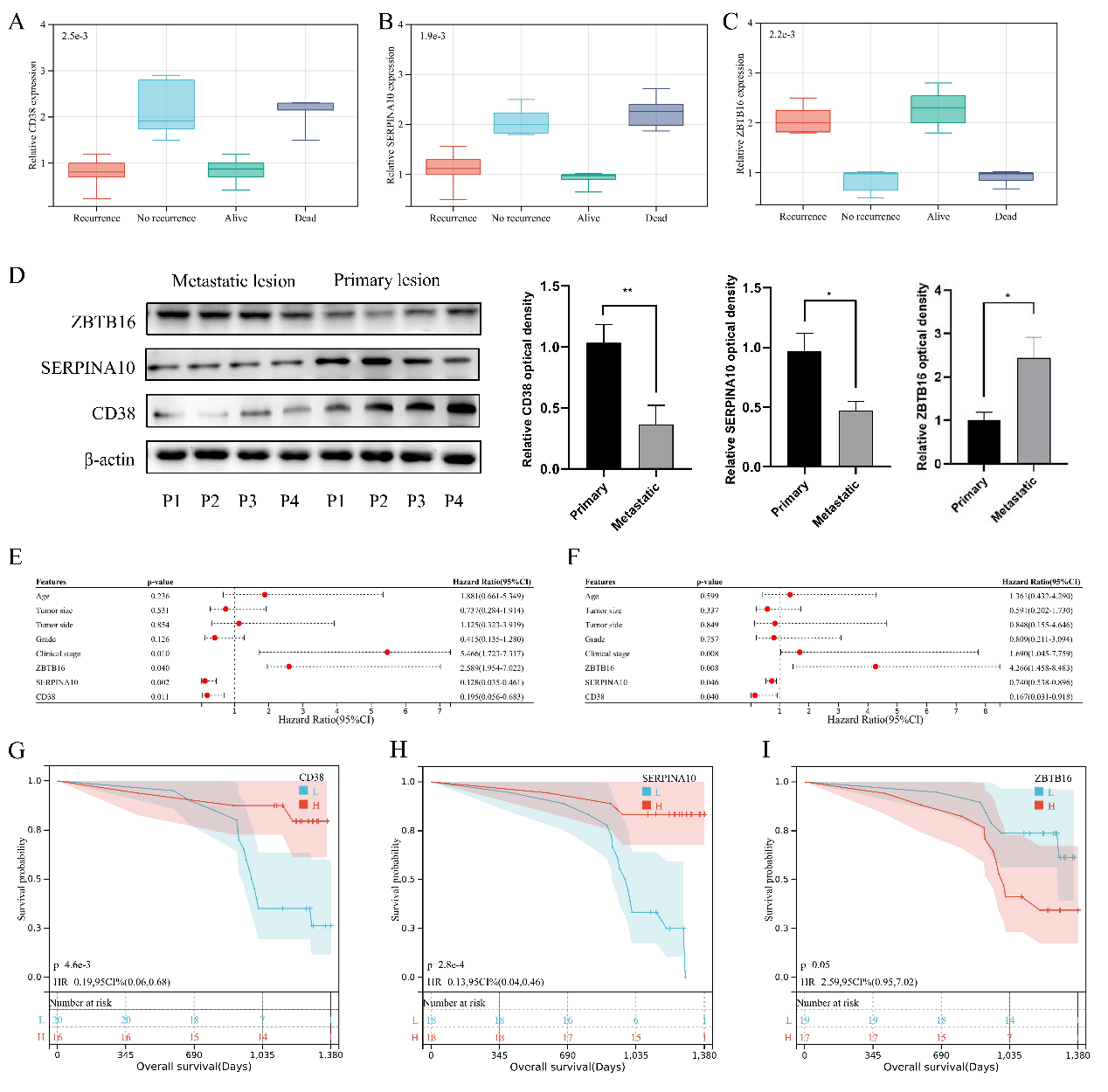

Supplement: Supplementary file 2 — Additional file 2: Figure S2. The coagulation-related genes could predict prognosis for ovarian cancer (OV) patients. The gene expression of (A) CD38, (B) SERPINA10, and (C) ZBTB16 in OV tissues, which was evaluated through the qRT-PCR analysis. (D) The Western blotting analysis showed the protein expression of CD38, SERPINA10, and ZBTB16 in primary and metastatic lesions from representative OV patients. The (E) univariate and (F) multivariate Cox regression analysis for OV patient survival, based on clinical features and three coagulation-related genes. The Kaplan–Meier (K–M) curves for OV patients, which were stratified by the expression of (G) CD38, (H) SERPINA10, and (I) ZBTB16. [file 12935_2023_3040_MOESM2_ESM.docx]
