# Supplementary material for: Clinical significance and immune infiltration analyses of a novel coagulation-related signature in ovarian cancer
Source: Cancer Cell Int. 2023 Oct 6;23:232. doi: 10.1186/s12935-023-03040-3 (PMC10559580; doi:10.1186/s12935-023-03040-3)
Supplement: Supplementary file 3 — Additional file 3: Table S3. The baseline characteristics of ovarian cancer (OV) patients. [file 12935_2023_3040_MOESM3_ESM.docx]

Supplement table 1. The baseline characteristics of ovarian cancer (OV) patients.

| **Features** | **Number of cases (n, %)** |
| --- | --- |
| Age |  |
| <60 years | 15 (41.67%) |
| ≥60 years | 21 (58.33%) |
| FIGO stage |  |
| I-II | 8 (22.22%) |
| III-IV | 28 (77.78%) |
| Pathological grade |  |
| G1-2 | 5 (13.89%) |
| G3 | 31 (86.11%) |
| Tumor size |  |
| <6 cm | 17 (47.22%) |
| ≥6 cm | 19 (52.78%) |
| Ascites |  |
| <1000ml | 18 (50.00%) |
| ≥1000ml | 18 (50.00%) |
| Survival status |  |
| Alive | 19 (52.78%) |
| Dead | 17 (47.22%) |
